# Supplementary material for: OpenMM 7: Rapid development of high performance algorithms for molecular dynamics
Source: PLoS Comput Biol. 2017 Jul 26;13(7):e1005659. doi: 10.1371/journal.pcbi.1005659 (PMC5549999; doi:10.1371/journal.pcbi.1005659)
Supplement: S1 Source Code — (ZIP) [file pcbi.1005659.s002.zip › OpenMM-7.0.1-Source/docs/api-c++/generated/OpenMM.SerializationProxy.html]

SerializationProxy — OpenMM @OPENMM\_MAJOR\_VERSION@.@OPENMM\_MINOR\_VERSION@ documentation


# SerializationProxy¶

*class* `OpenMM::``SerializationProxy`¶
:   A `SerializationProxy` is an object that knows how to serialize and deserialize objects of a particular type. This is an abstract class. Subclasses implement the logic for serializing particular types of logic.

    A global registry maintains the list of what `SerializationProxy` to use for each type of object. Call `registerProxy()` to register the proxy for a particular type. This is typically done at application startup or by a dynamic library’s initialization code.

    Methods

    |  |  |
    | --- | --- |
    | `SerializationProxy` | Create a new `SerializationProxy`. |
    | `~SerializationProxy` |  |
    | `getTypeName` | Get the name of the object type this proxy manipulates, as passed to the constructor. |
    | `serialize` | Subclasses implement this method to record information about an object being serialized. |
    | `deserialize` | Reconstruct an object from its serialized data. |

    `SerializationProxy`(*const* std::string &*typeName*)¶
    :   Create a new `SerializationProxy`.

        |  |  |
        | --- | --- |
        | Parameters: |  |

        - **typeName** – the name of the object type this proxy knows how to serialize. This name is stored in the output stream during serialization, and is used to select a proxy during deserialization. This typically is the class name, although that is not a requirement.

    `~SerializationProxy`()¶

    *const* std::string &`getTypeName`() *const*¶
    :   Get the name of the object type this proxy manipulates, as passed to the constructor.

    void `serialize`(*const* void \**object*, SerializationNode &*node*) *const* = 0¶
    :   Subclasses implement this method to record information about an object being serialized.

        |  |  |
        | --- | --- |
        | Parameters: |  |

        - **object** – a pointer to the object being serialized
        - **node** – all data to be serialized should be stored into this node, either directly as properties or indirectly by adding child nodes to it

    void \*`deserialize`(*const* SerializationNode &*node*) *const* = 0¶
    :   Reconstruct an object from its serialized data.

        |  |  |
        | --- | --- |
        | Parameters: |  |

        - **node** – a `SerializationNode` containing the object’s description

        |  |  |
        | --- | --- |
        | Returns: | a pointer to a new object created from the data. The caller assumes ownership of the object. |

    void `registerProxy`(*const* std::type\_info &*type*, *const* SerializationProxy \**proxy*)¶
    :   Register a `SerializationProxy` to be used for objects of a particular type.

        |  |  |
        | --- | --- |
        | Parameters: |  |

        - **type** – the type\_info for the object type
        - **proxy** – the proxy to use for objects of the specified type

    *const* SerializationProxy &`getProxy`(*const* std::string &*typeName*)¶
    :   Get the `SerializationProxy` to use for objects of a particular type, specified by name.

        |  |  |
        | --- | --- |
        | Parameters: |  |

        - **typeName** – the name of the object type to get a proxy for

    *const* SerializationProxy &`getProxy`(*const* std::type\_info &*type*)¶
    :   Get the `SerializationProxy` to use for objects of a particular type, specified by type\_info.

        |  |  |
        | --- | --- |
        | Parameters: |  |

        - **type** – the type\_info of the object type to get a proxy for

High performance molecular simulation on GPUs


### Navigation

- Core Objects
- Forces
- Integrators
- Extras

©2015, Stanford University and the Authors.
|
Powered by Sphinx 1.3.1
& Alabaster 0.7.7
|
Page source
